# Effectiveness of a Novel Temperature-Responsive Hydrogel (PF72) for Postoperative Pain Relief in Breast Augmentation

**DOI:** 10.3390/jcm13010110

**Published:** 2023-12-25

**Authors:** Jeongmok Cho, Ki Hyun Kim, Won Lee, Ju Young Go, Seong Hwan Kim

**Affiliations:** 1 Department of Plastic and Reconstructive Surgery, Etonne Plastic Surgery Clinic, Seoul 06531, Republic of Korea; niceview@gmail.com; 2 Department of Plastic and Reconstructive Surgery, Kangnam Sacred Heart Hospital, Hallym University College of Medicine, Seoul 07441, Republic of Korea; rlgus0608@hanmail.net; 3 Department of Plastic and Reconstructive Surgery, Yonsei E1 Plastic Surgery Clinic, Anyang 14046, Republic of Korea; e1clinic@daum.net; 4 Department of Plastic and Reconstructive Surgery, Atelier Plastic Surgery Clinic, Seoul 06584, Republic of Korea; ludinnight@hanmail.net

**Keywords:** breast augmentation, PF72, postoperative pain control

## Abstract

Breast augmentation can cause severe postoperative pain, leading to an increased length of hospital stay. Postoperative pain management following breast surgery has traditionally involved intravenous and oral analgesics. However, the misuse of opioids can cause severe complications. As a result, several alternative methods have been suggested. Fifty patients were included in this study. All the patients underwent dual-plane pocket dissection using the transaxillary approach and received smooth-type breast implants. The intervention group included 25 patients who received PF72 combined with ropivacaine, and the control group included 25 patients who received only bupivacaine. The Numerical Rating Scale (NRS) score was used to evaluate each patient’s pain. Three hours after the surgery, the average NRS scores of the control and intervention groups were 3.75 and 2.48, respectively. Six hours after the operation, the NRS scores of the control and intervention groups increased to 4.77 and 3.02, respectively. PF72 combined with ropivacaine was more effective than only bupivacaine for pain control in patients who underwent breast augmentations.

## 1. Introduction

Breast augmentation was one of the most frequently performed aesthetic surgical procedures in 2019 [1]. Breast implants have evolved over time. Currently, silicone gel-filled implants are commonly used [2,3]. Various surgical incisions, such as inframammary, periareolar, transaxillary, and transumbilical, have been employed during breast augmentation surgery [4]. The complications of breast augmentation procedures include bleeding, hematoma formation, infection, sensory changes, asymmetry, implant malposition, and capsular contractures [5]. Moreover, breast augmentation procedures can cause severe postoperative pain, nausea, and vomiting, and may be associated with wound dehiscence and an increased length of hospital stay [6]. Postoperative pain management following breast surgery has traditionally involved intravenous and oral analgesics [7]. Another modality might be epidural anesthesia [8]. However, overuse of opioids can cause abuse or addiction, and the misuse of opioids is a national public health crisis [9]. Moreover, with increased risks of epidural bleeding, severe infection, post-dural puncture headaches, subdural blockade and neurological dysfunction, thoracic epidurals were recently replaced with the current gold standard paravertebral blocks. Although safer than the thoracic epidural, some risks remain with paravertebral blocks, such as hypotension, vascular or accidental pleural puncture, and epidural or intrathecal placement [10]. Pain control is an important aspect that can affect a patient’s morbidity and satisfaction, which in turn can have an influence on experience and, further, personal recommendation of this ambulatory cosmetic procedure and the surgeon themselves. This has prompted the research on different analgesic alternatives, including intraoperative local anesthesia [7]. Therefore, multimodal analgesic pain management, including the use of local anesthetics at the incision site, peripheral nerve blocks, and intravenous administration of non-steroidal anti-inflammatory agents, has been suggested [11]. Among these modalities, local anesthetic agents are not commonly used because of their relatively short duration of action [12]. Bupivacaine irrigation of the breast pocket has been used recently for local anesthetic technique, but the effect of this is also known to be relatively short-lasting. Recently, a temperature-responsive hydrogel (PF72; TGel Bio, Inc., Ltd., Seoul, Republic of Korea) was developed as a sustained drug delivery device for reducing pain in the incisional area for 72 h postoperatively [13]. This newly developed hydrogel can be used to reduce pain after breast augmentation surgery. In this study, we performed breast augmentation with the administration of ropivacaine combined with PF72 and compared this approach with conventional pain control with bupivacaine irrigations using a retrospective hospital chart review.

## 2. Materials and Methods

### 2.1. Study Design and Patient Selection

In this retrospective observational study, all patients (aged 22–50 years) who underwent primary breast augmentation at a single center were enrolled for evaluation. Data for this study were obtained from the patients’ hospital charts. Patients with any previous surgery involving the breast, such as breast filler injection, fat graft, or mammotome; concomitant procedures such as nipple reductions, areolar reductions, and corrections of inverted nipples; concomitant drug use, such as neurologic drugs or antihypertensive drugs; and patients who had different-sized breast implants inserted were excluded. People with drug allergies to bupivacaine and ropivacaine were also excluded from this study. Patients were classified into two groups: the intervention group, which received PF72 combined with ropivacaine, and the control group, which received conventional pain control with bupivacaine irrigations. All patients were asked to report their pain scores, and these scores were compared between the two groups. This study was approved by the Hallym University Institutional Review Board (No. 2023-02-009). All patients provided consent for the publication of their data. All the procedures in this study were performed in accordance with the ethical standards of the institutional and national research committee and the Declaration of Helsinki.

### 2.2. Materials

**Figure 1:**
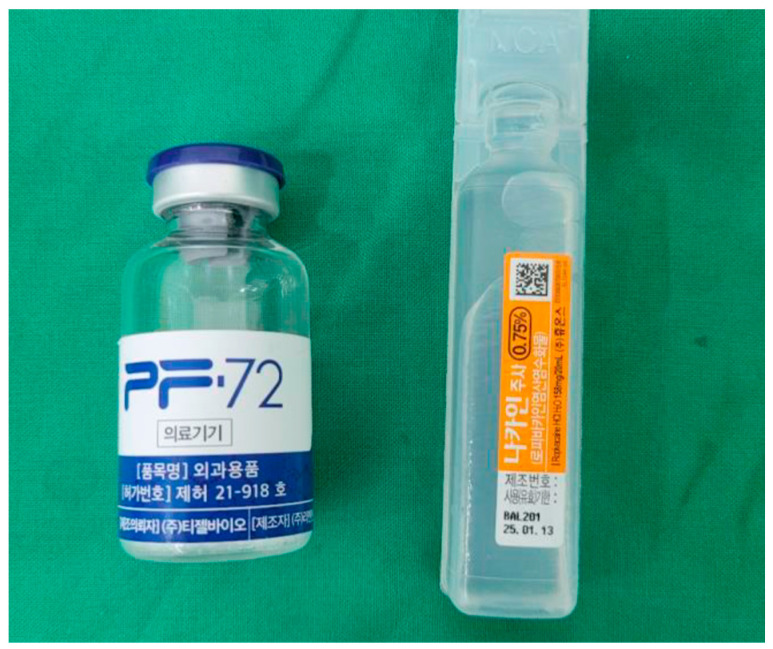
PF 72 and ropivacaine (Left PF 72 powder inside bottle (TGelBio, Seoul, Republic of Korea), Right ropivacaine solution 0.75% 20 mL (Seongnam, Republic of Korea)).

PF72 is provided in a vial containing lyophilized powders of Poloxamer 407 (20% *w*/*v*) and sodium hyaluronate (0.5% *w*/*v*) in sterile conditions [13]. It forms a viscous fluid when mixed with ropivacaine or bupivacaine at room temperature and turns into a gel at body temperature (Figure 1, Video S1). Ropivacaine is a drug used for a short duration of pain relief after administration (typically 2–6 h). It can be used in combination with PF72 to extend the duration of analgesia with the same amount of drug.

Poloxamer 407 has the property of changing shape with temperature. At low temperatures, it is liquid, but as the temperature rises, the Poloxamer 407 molecules twist around the sodium hyaluronate molecules and form a physical bond, or gel. This physical bond does not create a new substance; it only induces a physical change in form with no chemical change, with the other properties remaining the same. This can be verified using techniques such as the Fourier Transform Infrared (FTIR) spectra.

When PF72 is dissolved in ropivacaine 0.75% (ropipercarbine hydrochloride) and injected as a liquid into the painful area, the gel formed by body heat contains the drug (ropivacaine 0.75% [ropipercarbine hydrochloride]), and as the gel breaks down over time, the drug is slowly released, providing pain relief for as long as the drug is released.

### 2.3. Surgical Procedure

Under general anesthesia, patients were placed in the supine position. Axillary incisions were made for a transaxillary approach, and endoscopic dual-plane pocket dissections were performed. Before insertion of the breast implant, normal saline irrigation was performed twice with modified Adam’s solution (normal saline 500 mL + 10% betadine 50 mL + gentamicin 80 mg/2 mL + cefazolin 2 g) [14]. After breast pocket dissection was completed, the patient was seated upright on the operating bed, and the solution was inserted into the deepest space using a 17 G 120 mm cannula and 20-mL syringe. Opioid drugs were not used. No additional nerve blocks were performed other than the administration of local anesthesia to the incision site.

Patients in the control group (*n* = 25) received 2 ampules of bupivacaine (0.5%/20 mL) with 2 ampules of tranexamic acid (50 mg/5 mL). Patients in the intervention group (*n* = 25) received 2 ampules of tranexamic acid (50 mg/5 mL) in each breast pocket, followed by 1 ampule of PF72 mixed with ropivacaine (0.5%/20 mL). Thereafter, smooth-type breast implants were inserted. In the intervention group, 5 patients received Sebbin implants, 14 received Mentor implants, and 6 received Motiva implants. All patients had implants inserted bilaterally. Additionally, each patient’s breast implants were of the same volume.

### 2.4. Pain Evaluation

**Figure 2:**
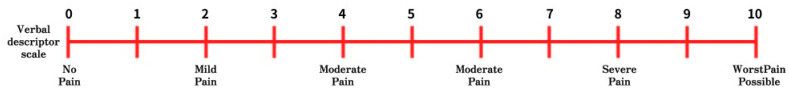
Eleven-point pain Numbered Rating Scale (NRS-11).

Postoperative pain was evaluated at 3, 6, 24, 48, and 72 h after the operation using the Numerical Rating Scale (NRS) score (Figure 2) [15].

0—Pain Free.

Mild Pain—Nagging pain, but does not really interfere with activities of daily living.

1—Pain is very mild, barely noticeable. Most of the time you do not think about it.

2—Minor pain. Annoying and may have occasional stronger twinges.

3—Moderate Pain—Interferes significantly with activities of daily living. Pain is noticeable and distracting; however, you can become accustomed to it and adapt.

4—Moderate pain– If you are deeply involved in an activity, it can be ignored for a period of time, but it is still distracting.

5—Moderately strong pain. It cannot be ignored for more than a few minutes, but with effort you still can manage to work or participate in some social activities.

6—Moderately strong pain that interferes with normal daily activities. Difficulty concentrating.

Severe Pain—Disabling; unable to perform activities of daily living.

7—Severe pain that dominates your senses and significantly limits your ability to perform normal daily activities or maintain social relationships. Interferes with sleep.

8—Intense pain. Physical activity is severely limited. Conversing requires great effort.

9—Excruciating pain. Unable to converse. Crying out and/or moaning uncontrollably.

10—Unspeakable pain. Bedridden and possibly delirious. Very few people will ever experience this level of pain.

### 2.5. Statistical Analyses

Differences between group means were compared using the Mann–Whitney U test, and *p*-values < 0.001 were considered statistically significant. All data were analyzed using IBM SPSS Statistics 28.0 (IBM Corp., Armonk, NY, USA) and reported as mean ± standard error.

## 3. Results

**Figure 3:**
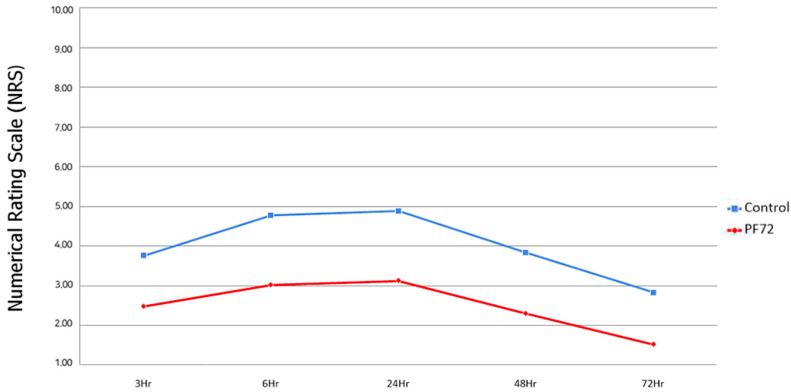
Average Numerical Rating Scale scores in the control and PF72 groups.

A total of 50 patients were included in this study. The average NRS values of patients in the control and PF72 groups are presented in Table 1 (Figure 3).

At 3 h after surgery (mean3H), the control group reported a mean NRS score of 3.78 ± 1.41, while the PF72 group reported a score of 2.48 ± 1.29. The difference in means was 1.30, with a standard error of 0.38 (*p* < 0.01).

At 6 h (mean6H), the control group had a mean NRS score of 4.74 ± 1.30, compared with a score of 3.02 ± 1.14 in the PF72 group. The difference in means was 1.72, with a standard error of 0.34 (*p* < 0.001).

At 24 h (mean24H), the mean NRS score was 4.90 ± 1.07 in the control group and 3.12 ± 1.17 in the PF72 group. The difference in means was 1.78, with a standard error of 0.31 (*p* < 0.001).

At 48 h (mean48H), the control group reported a mean NRS score of 3.82 ± 1.23, while the PF72 group reported a score of 2.30 ± 1.10. The difference between the groups was 1.52, with a standard error of 0.33 (*p* < 0.001).

Finally, at 72 h (mean72H), the control group had a mean NRS score of 2.86 ± 1.11, compared with a score of 1.52 ± 0.81 in the PF72 group. The difference in means was 1.34 with a standard error of 0.27 (*p* < 0.001).

In summary, the control group consistently experienced a significantly higher NRS score than the PF72 group across all time points, with the *p*-values indicating that the differences were statistically significant. There were no cases of cardiotoxicity in either group. Cardiac-related follow-up was not performed, and there were no cases of cardiovascular symptom complaints or treatments.

## 4. Discussion

As breast augmentations can cause severe pain, pain control is important to reduce possible complications. It is recommended to use more than two analgesics and administration routes to control pain in the early postoperative period [16]. Systemic analgesics, in combination with local anesthetic agents or regional nerve blocks, are usually used for pain control. When breast augmentation is performed, a local anesthetic agent is sprayed on the muscle or fascia area to reduce the pain sensation at the peripheral nerve endings. However, local anesthetic agents tend to have relatively short durations of action [17], and postoperative pain usually lasts for a few days. It is reported that uncontrolled acute-stage pain can develop into chronic pain. Continuous peripheral nerve blocks by catheter placement can be used for chronic pain control, although some adverse effects, such as patient dissatisfaction, technical problems, or overdose local exposure, might occur [18,19].

To prolong the effect of local anesthetic agents, multiple agents known as prolonged-duration local anesthesia were developed. DepoFoam^®®^ bupivacaine (Exparel, Pacira Pharmaceuticals) contains microscopic, spherical, and multivesicular liposomes with bupivacaine [20]. The use of biodegradable and injectable drug delivery devices is a viable option for more effective and safe management of acute postoperative pain during the first few days through sustained release of local anesthetic administered to the painful surgical wound site [13]. Nanoparticles of polyethylene glycol-co-polylactic acid used with 10% ropivacaine are reported to elicit 3–5 days of local anesthesia [21]. We chose to combine PF72 with ropivacaine rather than bupivacaine because of the known cardiotoxicity associated with bupivacaine, attributed to its cardiodepressant effect [22]. In contrast, ropivacaine has less cardiotoxicity due to its selective action. Although bupivacaine has the advantage of a longer duration of action, it is often more suitable for irrigation alone.

The use of PF72 is particularly advantageous due to its slow drug release. Furthermore, no signs of inflammatory reactions to the PF72 hydrogel were observed in any animals used in 3 in vivo tests, indicating excellent biocompatibility of the hydrogel as shown in previous studies [23]. The results of some studies showed excellent safety and effective long-term pain relief of ropivacaine administered using PF72 [13]. The feature of PF-72′s slow drug release compensates for the short duration of action typically associated with local anesthetics such as ropivacaine. By combining ropivacaine with PF72, we are taking advantage of both the prolonged drug release of PF72 and the low cardiotoxicity profile of ropivacaine. This strategic combination aims to maximize pain relief efficacy while minimizing potential adverse cardiovascular effects. Especially regarding the onset time, since bupivacaine’s onset is a little slower, ropivacaine is more advantageous for the drug to be effective more quickly when used along with the slow-released PF72. Thus, ropivacaine was chosen because it creates a higher potency and longer-lasting local anesthesia when mixed with PF72. 

In our study, the NRS score in the PF72 group was 1.30–1.78 points lower than that of the control group. When undergoing breast augmentation, patients are typically unable to feel much pain immediately after the operation. Patients’ pain tends to improve after 2 days. Our study found that, 6 h after surgery, the PF72 group showed an NRS score 1.72 points lower than that of the control group. Twenty-four hours after surgery, the PF72 group showed an NRS score 1.78 points lower than that of the control group. It is important to assess patients’ pain by numerical scores, although the qualitative description of the NRS scores should also be considered. In the PF72 group, the highest score was 3.12 24 h after surgery. An NRS score of 3 is considered to indicate mild pain, described as “pain is noticeable and distracting; however, you can get used to it and adapt.” When assessing the pain of the control group 24 h after surgery, the NRS was almost 5. An NRS score of 5 is considered moderate stinging pain and is described as “it cannot be ignored for more than a few minutes, although with effort, you can still manage to work or participate in some social activities.” The difference of 2 points in the NRS scores appears insignificant, although the actual pain and resulting limitation of daily activities between the two scores are very different.

An advantage of using this pain control gel is that it is convenient for use during breast surgery. When using bupivacaine irrigation, the liquid tends to spill out into the axillary incision area. However, since PF72 is in gel form, it is more likely to stay in the breast pocket. 

Our study results indicated better pain control with the use of PF72 compared to standard treatment with bupivacaine irrigation. However, our study has the following limitations.

Inter-patient variability: Individual patients may have responded differently to treatment, and this could be influenced by factors such as individual pain thresholds, drug sensitivity and general health status. Thus, this variability may introduce potential confounding variables and make it difficult to generalize the results to a wider population.

Comparative study design: There may be potential limitations related to study design. To overcome inter-patient variability, it is proposed to compare bupivacaine in one breast and PF72 in the other breast in the same patient. While this design aims to control for individual differences to some extent, it introduces the challenge of ensuring symmetry between the two breasts, and there may still be anatomical or physiological differences between the breasts that could affect the results.

Short duration of observation: Our study only assessed patients at 3, 6, 24, 28, and 72 h after surgery. It may not capture the full range of pain experiences or potential complications that may occur beyond the initial postoperative period.

Single-center study: Our study is conducted at a single center, which may limit the generalizability of the findings to a broader population, as patient demographics and surgical practices may vary between institutions.

Subjective nature of pain assessment: We relied on NRS scores to assess pain, which is a subjective measure. Although the NRS is a commonly used tool, it is subject to individual interpretation and factors such as patient expectations; psychological factors may also influence pain perception.

Generalizability: This study may not account for the diversity of patient populations or surgical techniques used in different settings. The findings may be specific to the population and procedures studied, limiting their applicability to a more diverse patient population or different surgical approaches.

It is important to note that these limitations do not invalidate the results of this study, but rather highlight areas where future research or additional considerations may be needed to more fully understand the efficacy and safety of PF72 in breast augmentation surgery.

## 5. Conclusions

The results of this study indicated that the PF72 and ropivacaine combination provided superior pain control compared to the conventional bupivacaine irrigation method. The NRS scores consistently favored the PF72 group, indicating lower pain levels at various postoperative time points (3, 6, 24, 48, and 72 h). The differences in NRS scores were statistically significant, demonstrating the potential effectiveness of the PF72 and ropivacaine combination in reducing postoperative pain.

The use of PF72 combined with ropivacaine could be a promising alternative for postoperative pain management in breast augmentation procedures. Future studies with a larger sample size, multi-center design, longer follow-up duration, and potentially more diverse patient populations are warranted to confirm and expand upon these initial findings. Additionally, investigating potential complications, patient satisfaction, and long-term outcomes would contribute to a more comprehensive understanding of the benefits and risks associated with this novel pain control approach in breast augmentation surgery.

## Figures and Tables

**Table 1:** Average Numerical Rating Scale scores in the control and PF72 groups.

	Control (*n* = 25)	PF72 (*n* = 25)	Pain Difference (SEΔ **)	*p*-Value
Mean3H *	3.78 ± 1.41	2.48 ± 1.29	1.30 (0.38)	0.002
Mean6H *	4.74 ± 1.30	3.02 ± 1.14	1.72 (0.34)	<0.001
Mean24H *	4.90 ± 1.07	3.12 ± 1.17	1.78 (0.31)	<0.001
Mean48H *	3.82 ± 1.23	2.30 ± 1.10	1.52 (0.33)	<0.001
Mean72H *	2.86 ± 1.11	1.52 ± 0.81	1.34 (0.27)	<0.001

* Data are reported as mean ± standard error. Data were compared using the Mann–Whitney U test. ** Standard Error with a change or difference.

## Data Availability

Data are contained within the article.

## Supplementary Materials

The following supporting information can be downloaded at https://www.mdpi.com/article/10.3390/jcm13010110/s1. Video S1: Morphology of PF72. PF72 is provided in a sterile condition as lyophilized powders of Poloxamer 407 (20% *w*/*v*) and sodium hyaluronate (0.5% *w*/*v*), demonstrating a physiologically clean state.
